# Inclusion of Clinicians in the Development and Evaluation of Clinical Artificial Intelligence Tools: A Systematic Literature Review

**DOI:** 10.3389/fpsyg.2022.830345

**Published:** 2022-04-07

**Authors:** Stephanie Tulk Jesso, Aisling Kelliher, Harsh Sanghavi, Thomas Martin, Sarah Henrickson Parker

**Affiliations:** ^1^Fralin Biomedical Research Institute, Virginia Tech, Roanoke, VA, United States; ^2^Institute for Creativity, Arts, and Technology, Blacksburg, VA, United States; ^3^Department of Computer Science, College of Engineering, Virginia Tech, Blacksburg, VA, United States; ^4^Carilion Clinic, Roanoke, VA, United States; ^5^Department of Electrical and Computer Engineering, College of Engineering, Virginia Tech, Blacksburg, VA, United States; ^6^Department of Health Systems and Implementation Science, Virginia Tech Carilion School of Medicine, Roanoke, VA, United States

**Keywords:** artificial intelligence (AI), clinical AI, machine learning, clinician, human-centered design, evaluation, healthcare

## Abstract

The application of machine learning (ML) and artificial intelligence (AI) in healthcare domains has received much attention in recent years, yet significant questions remain about how these new tools integrate into frontline user workflow, and how their design will impact implementation. Lack of acceptance among clinicians is a major barrier to the translation of healthcare innovations into clinical practice. In this systematic review, we examine when and how clinicians are consulted about their needs and desires for clinical AI tools. Forty-five articles met criteria for inclusion, of which 24 were considered design studies. The design studies used a variety of methods to solicit and gather user feedback, with interviews, surveys, and user evaluations. Our findings show that tool designers consult clinicians at various but inconsistent points during the design process, and most typically at later stages in the design cycle (82%, 19/24 design studies). We also observed a smaller amount of studies adopting a human-centered approach and where clinician input was solicited throughout the design process (22%, 5/24). A third (15/45) of all studies reported on clinician trust in clinical AI algorithms and tools. The surveyed articles did not universally report validation against the “gold standard” of clinical expertise or provide detailed descriptions of the algorithms or computational methods used in their work. To realize the full potential of AI tools within healthcare settings, our review suggests there are opportunities to more thoroughly integrate frontline users’ needs and feedback in the design process.

## Introduction

The development and use of artificial intelligence (AI) in healthcare contexts has the potential to greatly improve the delivery and practice of medicine (Sim et al., 2001), yielding benefits for patients and clinicians (Chute and French, 2019; Bates et al., 2020; Brice and Almond, 2020; Sendak et al., 2020). The use of AI in medicine can assist clinicians and organizations with a desirable shift toward evidence-based adaptive healthcare (Sackett et al., 1996; Fineout-Overholt et al., 2005). Clinical Decision Support Systems (CDSS) integrate AI and machine learning (ML) algorithms to support decision making in domains such as diagnosis (Beede et al., 2020; McKinney et al., 2020) and treatment planning (Jin et al., 2020; Jacobs et al., 2021). Clinical AI is currently proposed across multiple medical domains addressing issues such as clinician burnout (Arndt et al., 2017; Ash et al., 2019), medical errors (Cai et al., 2019a; Van Camp et al., 2019), and detecting frequently unrecognized and life-threatening conditions such as sepsis (Sendak et al., 2020). However, while a growing body of research literature describes the promise of these algorithmically driven approaches, there remains a paucity of evidence demonstrating sustained successful integration of AI into clinical practice (Middleton et al., 2016; Osman Andersen et al., 2021).

There are two major challenges to successful integration of AI into clinical practice. One challenge for integration is the translation of technologies and methods from research domains into ecologically valid clinical practice (Wears and Berg, 2005; Schriger et al., 2017; Sligo et al., 2017; Yang et al., 2019; Beede et al., 2020; Li et al., 2020; Schwartz et al., 2021; Wong et al., 2021). Specifically, the design and implementation of clinical AI tools are mismatched with the actual context of clinical work or the true needs of frontline clinical users (Khairat et al., 2018; Yang et al., 2019; Jacobs et al., 2021). Unfortunately, even if the technology functions correctly, it may not get used if it does not match the clinical workflow (Sittig et al., 2008). The second pervasive challenge is that technical functionality of many computational models are not validated against clinician expertise (i.e., the “gold standard”) through a direct comparison of a clinician’s and model’s performance on the same task (Schriger et al., 2017; Shortliffe and Sepúlveda, 2018; Schwartz et al., 2021), which is necessary to ensure that tools provide value to the clinicians who are asked to use them. Addressing these concerns is challenging given the complexity of clinical work in the real-world and the tendency of research to occur in academic silos (Sendak et al., 2019; Asan and Choudhury, 2021), which results in a significant gap in current literature. However, it is critically important to ensure that clinical AI tools are not disruptive and add value to clinical practice (Sujan et al., 2019; Choudhury and Asan, 2020). Additional research is needed to understand how to address these broad limitations and to determine best practices for clinical AI deployment to maximize usage across domains of care (Sligo et al., 2017; Shortliffe and Sepúlveda, 2018; Jacobs et al., 2021; Osman Andersen et al., 2021).

Human-centered design (HCD), or the philosophy that understanding human needs, capabilities, and behaviors must come first in the design process (Norman, 2002), provides a methodological approach for the development of clinical AI tools that can overcome the translational gap (Khairat et al., 2018; Shortliffe and Sepúlveda, 2018; Wiens et al., 2019). This form of design emphasizes a dynamic and iterative process involving the identification of application stakeholders and their needs, the development of design prototypes, and the evaluation of products by end-users (Norman, 2002, 2005). Recent work emphasizes the potential for designers to create new tools, methods, and design processes to more adeptly handle AI and machine learning as fundamental (but not exclusive) materials within the design process (Holmquist, 2017; Kelliher et al., 2018; Yang et al., 2018). When designers adopt a user-centered approach and engage with a variety of stakeholders (including clinicians) in the early stages of development, the final products are typically designed to fit specific clinical needs and may be better oriented for acceptance (Cai et al., 2019a,b) and success (Mamlin et al., 2007; Kujala, 2010; Wiens et al., 2019). The involvement of end-users in all, or at least some of the design, implementation, and evaluation process can also engender higher levels of trust and appropriate trust calibration in clinical AI tools, encouraging tool use (Tcheng et al., 2017) and confidence in application output (Chen, 2018; Benda et al., 2021). However, designing for clinical contexts using a HCD approach also presents clear challenges including issues of access to physical spaces and/or digital records (Kulp and Sarcevic, 2018; Yang et al., 2019), the inability of design teams to iterate across multiple design cycles (Middleton et al., 2016; Osman Andersen et al., 2021) and issues with incomplete or even no substantive evaluations carried out by the design team (Coyle and Doherty, 2009).

Overall, there is a need to develop better standards for the design, implementation and evaluation of clinical AI tools to ensure that they provide value to their intended clinical end users (Wiens et al., 2019; Li et al., 2020). To support this need, the aim of this article is to survey the current peer-reviewed literature detailing the implementation of AI tools into healthcare, with particular emphasis on how frontline clinicians were engaged in the implementation process. Specifically, we aim to identify (1) how designers and developers of clinical AI interact with clinical end users during the design and implementation process, and (2) how designers and developers evaluate the value of their products to clinicians once they are implemented into clinical practice. This work extends that of other recent reviews (Middleton et al., 2016; Asan and Choudhury, 2021; Schwartz et al., 2021) and focuses on clinicians to present a comprehensive picture of the methods employed by tool designers to understand and report on clinician needs surrounding clinical AI tools.

## Methods

**FIGURE 1:**
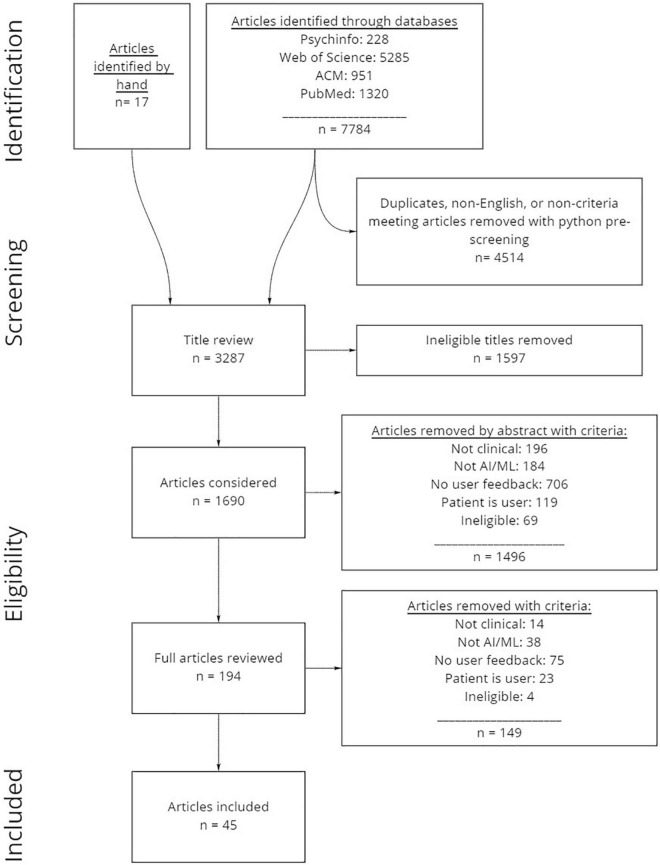
Systematic review process and numbers of articles included and excluded. Articles were first identified through online databases, then pre-processed using python scripts. Next, all articles were manually reviewed by title and abstract prior to reviewing full articles to evaluate which articles were included.

A systematic, multistep literature review was conducted in line with PRISMA guidelines for systematic reviews (see Figure 1 for complete steps and numbers). We note that while it is likely that many clinical AI efforts are not described in published literature, this review seeks to establish a broad understanding of the kinds of design efforts undertaken (i.e., what types of AI products are being developed and for which domains), and the design and user evaluation processes enacted.

### Identification and Screening

Our identification approach included a systematic database and journal search of PsycInfo, Web of Science (includes Medline, NeurIPS, AAAI, and IEEE), ACM, and PubMed. We selected these sources to capture a broad range of research related to healthcare, AI and ML, and user or human centered research. The database search process was conducted in three separate batches. The first batch (batch 1, *n* = 1959) was retrieved on 11/2/2020, and included all articles published from 1/1/2015 onward. This timeframe was selected due to the dramatic increase of articles during this 5-year timeframe. A further two batches of articles were retrieved (batch 2, *n* = 2369 added; pulled 11/18/2021), and (batch 3, *n* = 4218 added; total *n* = 7784, excluding duplicates from same sources; pulled 4/5/2021) which identified additional articles published after the dates of the first and second searches. The search criteria (Table 1) were selected to capture a broad range of results, but also to reduce the total number of ineligible records thus ensuring that the authors had the capability of screening and reviewing all identified records. Seventeen additional references were discovered through hand search.

**TABLE 1:** Three categories of search criteria used to identify articles.

Clinical domain terms	“Decision support,” “healthcare,” “health care,” “physician,” “patient,” “clinic*” (e.g., clinical, clinician), “nurs*” (e.g., nurse, nursing), “diagnosis,” “medical records” (e.g., electronic medical records), or “health records” (e.g., electronic health records)
AI terms	“AI,” “ML,” “machine learning,” “deep learning,” “intelligen*” (e.g., intelligent sensors), “ambient” (e.g., ambient awareness or ambient intelligence), “CNN,” “RNN,” “neural network,” “convolutional,” “recurrent,” “Markov” (e.g., Hidden Markov Model), “reinforcement learning,” “SVM,” “support vector”
User feedback terms	“UX,” “usability,” “user” (e.g., user test, user centered design), “adoption” (e.g., technology adoption), “human centered,” HCI, “human computer” (e.g., human computer interaction), “human AI” (e.g., human AI interaction)

The asterisks (“*”) denotes a truncation to include variant endings of related words (e.g., “nurs*” can flag results including “nurse”, “nurses”, and “nursing”).

We used python scripts to compile and pre-screen the articles extracted from databases sources. The Selenium web scraping package (Salunke, 2014) assisted in the extraction of records from databases that did not allow for simple BibTeX or csv downloads. We created custom scripts to compile all records into the same csv format, then screened for duplicate titles using the Levenshtein python package (Deveopedia, 2019), and screened out articles not written in English and articles that did not include any of the search criteria in titles, abstracts, or key terms (*n* = 4514), resulting in *n* = 3287 remaining articles. Finally, if the word “review” was included in the title, abstract or key terms, the record was marked to assist in the manual screening process.

After the python pre-screening process, titles were manually reviewed to remove anything that was ineligible due to irrelevance to the topic (e.g., topics outside of the realm of human health such as zoology or data security, public health research, articles describing biochemical or pharmaceutical research that might occur in a medical lab, review articles, theses, and dissertations; see Table 2 for a definition). After reviewing titles, *n* = 1597 ineligible articles were removed, resulting in *n* = 1682 remaining articles for manual abstract review.

**TABLE 2:** Evaluation criteria used for inclusion and exclusion.

NA = no AI	Included studies needed to include some type of AI/ML, or the authors themselves needed to explicitly related their research to AI with or without the addition of algorithms. The assignment of the code “NA” meant that there was no machine learning or artificial intelligence involved in the study, nor did the authors claim that the study was related to AI. For instance, while a decision tree algorithm and predictive analytics are not technically AI, if the article reports any algorithm to be AI and asks clinicians about AI tools, we considered this to be AI. Additionally, hypothetical AI/ML technologies were not excluded

NC = not clinical	Included articles needed to focus on challenges and work within a clinical domain. The assignment of the code “NC” meant that the article was not focused on the support of clinicians in clinical contexts. While diagnostic tests and tools were relevant, research focused on the work of lab technicians, speeding up lab results, or aiding in the process of quality improvement were excluded. Community/public health research efforts were also excluded
NU = no user feedback	Included articles needed to include some form of explicit feedback from intended clinical end users regarding a proposed or existing tool, or about AI/ML in general. The assignment of the code “NU” meant that the article did not describe any attempt to observe what clinicians thought about AI/ML and/or a specific clinical AI tool. If the users’ opinions are considered in any stage, the article could be included (for instance, interviews or committees of users to determine what users want prior to creating the system, or even informal feedback from users at the end of an evaluation). Efforts that used “user tests” solely for the purpose of validating system performance and which did not include any report of user opinions were excluded
PU = a patient is the user	Included articles needed to focus on clinicians as end users. The assignment of the code “PU” meant that patients were the intended users and clinicians were not considered to be primary users of any component of the tool/system. Articles that did include clinicians and patients as users of different components of the design were not excluded
Ineligible	Articles that were considered ineligible included research that was outside of the realm of human health (e.g., zoology, data security), or were related to public health research (e.g., tracking the spread of HIV, measuring depression and anxiety on social media), articles that described the technical details of laboratory tests (e.g., new biochemical assays), articles that did not present primary research (e.g., published study protocols, case studies, review articles, editorials, or position pieces), and papers that were not peer-reviewed (e.g., published theses or dissertations)

### Consensus and Eligibility

After the initial screening process, two independent raters (STJ and HS) determined article eligibility through manual abstract review. For inclusion, articles had to describe primary research that (1) was related to AI/ML by involving any algorithm purported to be AI/ML by authors, (2) considered clinicians as primary users and focused on use within a clinical context, and (3) described the collection of some form of explicit user feedback. The inclusion/exclusion criteria codes and definitions are presented in Table 2. To evaluate consensus, the raters worked individually to review the abstracts from 20% of the articles from batch 1 (*n* = 95 articles). The initial consensus was 67% agreement on which articles to include and exclude. To establish greater consensus, the raters reviewed a subset of the coded articles together and discussed why individual coding decisions were made. Following the consensus building exercise, each rater independently re-reviewed the original 95 article abstracts with access to their own and their co-rater’s codes to re-evaluate consensus. Final consensus on these articles was 83% agreement on which articles to include. The raters divided up the remaining articles from batch 1 (*n* = 369 remainder), with 10% (*n* = 37) randomly selected for both raters to review to test consensus again, which resulted in a final acceptable consensus of 84% on which articles to include and exclude. When there was disagreement, articles were included for full article review where a better determination could be made. The raters then worked independently to review all remaining abstracts. In total, *n* = 1496 articles were removed, and *n* = 194 articles were considered for a full article review.

During the full article review process, the two raters divided up the remaining *n* = 194 articles and determined eligibility using the same inclusion/exclusion criteria presented in Table 2. Upon completion, a total of *n* = 148 articles were removed yielding a final total of *n* = 45 articles that were included.

### Data Extraction and Analysis

If an article met inclusion criteria during the full article review process, the rater then reviewed the article and filled out a Google form that was created to capture and organize the information that was determined to be of interest. This was an iterative process, where entry fields within the Google form were adjusted or added as the articles were being reviewed, resulting in re-review and re-categorization of data to synthesize a. The final dataset included details related to the type of study that was being presented, the methods being employed, the individuals whose perspectives were being studied (i.e., users and other stakeholders) and when they were engaged relative to design progress, the types of tools that were being developed, details on the underlying algorithms being used, and whether or not the article reported any insights into clinician and stakeholder trust of clinical AI tools, which is presented below in the section “Results.”

## Results

### Reviewed Articles and Product Matrix

While our original intention was to examine articles that described the design of novel clinical AI tools and the process of gathering feedback from clinical end users, we identified other types of articles that met our inclusion criteria. The 45 included articles can broadly be characterized as comprising four primary categories (see “Type of study” columns in Table 3):

(1) *A design study*, defined as the design and study of a novel clinical AI tool. These included efforts that used a user-centered approach and consulted end users early and throughout the design process as well as efforts which primarily focused on the description of the algorithm and application and reported at least minimal user feedback, typically at the end of the design or implementation process (*n* = 24, see rows in gray in Table 3).
(2) *A third party study*, defined as research conducted on clinical AI tools (i.e., the tool design team was not responsible for the publication) to understand the effect of implementation, what end users thought of the product(s), or what end users would need from the product for a successful implementation (*n* = 4).
(3) *A preliminary design study* defined as preliminary user-centered research to collect feedback prior to the development of a clinical AI tool (*n* = 6).
(4) *Empirical research* to evaluate clinical end user experiences, needs, or concerns about hypothetical clinical AI tools or in general (*n* = 10).

**TABLE 3:** Product matrix of clinical AI tool literature reviewed.

		Type of study				Real tool?		Method type		Method(s)											Metric(s)							Stakeholders			Target users						User consult time					Algorithm(s)						Validation			Trust?	
	References	Design study	3rd party study	Preliminary design study	Empirical research	Real tool	Hypothetical tool	Qualitative	Quantitative	Co-creation	Context analysis	Ethnography/observations	Focus groups	Interviews	Iterative design	Survey	User evaluations	Validation/performance	Other	Total Participants	Preferences	Performance	Errors	Empirical observation	Trust	Opinions	Other	Clinicians	Patients	Other	Nurses	Physicians	Clinicians (in general)	Specific clinical specialties	Patients	Other	Beginning	Before or during prototype	Middle or iterative design	At the end of research effort	Not a design study	Deep learning	Regression	Described specific algorithm	Generic description	Named product	Hypothetical (research)	Validated here or elsewhere	Accuracy only	None, or non-design	Yes	No
Diagnosis	Umer et al., 2019 *	✓				✓		✓	✓	✓				✓	✓		✓		✓	6	✓	✓						✓				✓					✓	✓	✓					✓				✓				✓
	Beede et al., 2020	✓				✓		✓				✓		✓						18				✓				✓	✓				✓			✓	✓			✓		✓			✓				✓		✓	
	Ehtesham et al., 2019	✓				✓		✓	✓							✓	✓	✓		Unknown	✓	✓				✓	✓	✓				✓		✓						✓				✓					✓			✓
	Xu et al., 2018	✓				✓		✓	✓					✓	✓		✓			22		✓	✓			✓		✓				✓						✓		✓		✓								✓	✓	
	Khodabandehloo et al., 2021 *	✓				✓		✓	✓					✓		✓				8		✓				✓		✓	✓			✓			✓					✓			✓	✓		✓		✓			✓	
	Cai et al., 2019a	✓				✓		✓	✓						✓		✓			15	✓	✓			✓	✓		✓				✓					✓	✓	✓	✓		✓				✓		✓			✓	
	Sakellarios et al., 2020 *	✓				✓		✓	✓							✓	✓			95		✓				✓		✓				✓		✓				✓		✓						✓		✓				✓
	Tschandl et al., 2020	✓				✓		✓	✓							✓				302		✓	✓		✓			✓	✓			✓								✓		✓						✓			✓	
	Trivedi et al., 2018 *	✓				✓			✓							✓		✓		9	✓						✓	✓					✓				✓			✓				✓		✓		✓				✓
	Wang et al., 2021 *		✓			✓		✓			✓	✓		✓						22				✓		✓		✓				✓									✓					✓				✓		✓
	Strohm et al., 2020		✓			✓		✓						✓					✓	24				✓		✓		✓		✓		✓		✓							✓					✓				✓	✓	
	Ltifi et al., 2020			✓			✓		✓							✓	✓		✓	30		✓				✓		✓					✓						✓								✓			✓		✓
	Bourla et al., 2020 *				✓		✓		✓							✓			✓	2322	✓				✓	✓		✓			✓										✓						✓			✓	✓	
	Blease et al., 2019 *				✓		✓	✓	✓							✓				720						✓		✓				✓									✓						✓			✓		✓
	Cai et al., 2019b				✓	✓	✓	✓						✓			✓			21	✓					✓		✓				✓									✓	✓								✓	✓	
	Waymel et al., 2019				✓		✓		✓							✓				617	✓					✓		✓				✓									✓						✓			✓		✓
	Okolo et al., 2021				✓		✓	✓						✓					✓	21						✓		✓					✓								✓						✓			✓	✓	
	Botwe et al., 2021				✓		✓	✓								✓				1020					✓	✓		✓					✓								✓						✓			✓		✓

Treatment planning	Umer et al., 2019 *	✓				✓		✓	✓	✓				✓	✓		✓			6	✓	✓						✓				✓					✓	✓	✓							✓		✓				✓
	Jin et al., 2020 *	✓				✓		✓	✓					✓					✓	9		✓				✓		✓			✓						✓		✓			✓								✓	✓	
	Lee et al., 2020 *	✓				✓		✓	✓	✓		✓				✓	✓	✓		16	✓				✓	✓		✓	✓			✓		✓			✓	✓	✓			✓		✓				✓			✓	
	Kumar et al., 2020	✓				✓		✓	✓							✓	✓			43		✓				✓	✓	✓				✓								✓				✓				✓				✓
	Nemeth et al., 2016	✓				✓		✓		✓		✓		✓			✓		✓	151				✓	✓	✓		✓		✓			✓				✓	✓	✓	✓					✓					✓	✓	
	Sakellarios et al., 2020 *	✓				✓		✓	✓							✓	✓			95		✓				✓		✓				✓		✓				✓		✓						✓		✓				✓
	Torrents-Barrena et al., 2019	✓				✓		✓	✓							✓	✓			5		✓	✓		✓	✓		✓	✓			✓		✓						✓		✓		✓				✓			✓	
	Benrimoh et al., 2021	✓				✓		✓	✓							✓	✓		✓	51					✓	✓	✓	✓	✓			✓					✓	✓				✓			✓			✓			✓	
	Wang et al., 2021 *		✓			✓		✓			✓	✓		✓						22				✓		✓		✓				✓									✓					✓				✓		✓
	Jacobs et al., 2021			✓			✓	✓					✓	✓					✓	10					✓	✓		✓				✓					✓	✓									✓			✓	✓	
	Yang et al., 2016			✓			✓	✓				✓		✓						24					✓	✓		✓				✓	✓	✓			✓										✓			✓	✓	
	Yang et al., 2019			✓			✓	✓				✓		✓						17					✓	✓		✓				✓		✓			✓	✓									✓			✓		✓
	Blease et al., 2019 *				✓		✓	✓	✓							✓				720						✓		✓				✓									✓						✓			✓		✓

Risk assessment	Jin et al., 2020 *	✓				✓		✓	✓					✓					✓	9		✓				✓		✓			✓						✓		✓			✓								✓	✓	
	Brennan et al., 2019	✓				✓		✓	✓							✓	✓	✓		20		✓				✓		✓				✓		✓						✓					✓	✓			✓			✓
	Sandhu et al., 2020	✓				✓		✓						✓						15						✓		✓					✓							✓		✓						✓			✓	
	Sendak et al., 2020	✓				✓		✓	✓			✓			✓	✓	✓		✓	Unknown	✓	✓	✓	✓	✓	✓		✓		✓	✓	✓					✓	✓	✓	✓		✓						✓			✓	
	Lagani et al., 2015	✓				✓		✓	✓										✓	2						✓		✓	✓				✓							✓			✓	✓				✓				✓
	Jauk et al., 2021		✓			✓		✓	✓							✓			✓	47		✓				✓		✓			✓	✓									✓			✓						✓		✓
	Gilbank et al., 2020			✓			✓	✓						✓	✓				✓	10					✓	✓		✓		✓		✓						✓	✓								✓			✓	✓	
	Bourla et al., 2020 *				✓		✓		✓							✓			✓	2322	✓				✓	✓		✓			✓										✓						✓			✓	✓	
	Baxter et al., 2020				✓		✓	✓						✓					✓	15						✓		✓		✓			✓			✓					✓						✓			✓	✓	

Ambient intelligence and tele monitoring	Cohen et al., 2017	✓				✓		✓	✓				✓	✓		✓				17						✓		✓			✓									✓					✓					✓		✓
	Lee et al., 2020 *	✓				✓		✓	✓	✓		✓				✓	✓	✓		16	✓				✓	✓		✓	✓			✓		✓			✓	✓	✓			✓		✓				✓			✓	
	Khodabandehloo et al., 2021 *	✓				✓		✓	✓					✓		✓				8		✓				✓		✓	✓			✓		✓	✓					✓			✓			✓		✓			✓	
	Bourla et al., 2020 *				✓		✓		✓							✓			✓	2322	✓				✓	✓		✓			✓										✓						✓			✓	✓	
	Bourbonnais et al., 2019				✓		✓	✓			✓			✓						20						✓		✓	✓	✓			✓			✓					✓						✓			✓	✓	
	Poncette et al., 2020				✓		✓		✓							✓				270	✓				✓	✓		✓			✓	✓									✓						✓			✓	✓	
	Bourla et al., 2018				✓		✓		✓							✓			✓	515						✓		✓				✓		✓							✓						✓			✓		✓

NLP	Long et al., 2016 *	✓				✓		✓	✓					✓		✓	✓			12			✓			✓	✓	✓	✓				✓		✓					✓					✓					✓		✓
	Trivedi et al., 2019	✓				✓		✓	✓					✓		✓		✓		15		✓				✓	✓	✓		✓		✓								✓				✓				✓				✓
	Trivedi et al., 2018 *	✓				✓			✓							✓		✓		9	✓						✓	✓					✓				✓			✓						✓		✓				✓
	Petitgand et al., 2020		✓			✓		✓			✓	✓		✓						20				✓		✓		✓		✓		✓									✓				✓					✓	✓	
	Goss et al., 2019				✓		✓		✓							✓				1731	✓							✓												✓							✓			✓		✓

Administrative tasks	Donoso-Guzmán and Parra, 2018	✓				✓		✓								✓		✓		24	✓	✓					✓	✓				✓								✓				✓				✓				✓
	Long et al., 2016 *	✓				✓		✓	✓					✓		✓	✓			12			✓			✓	✓	✓	✓				✓		✓					✓					✓					✓		✓
	Klakegg et al., 2018	✓				✓		✓	✓					✓			✓	✓		19		✓	✓			✓		✓			✓							✓		✓				✓					✓			✓
	Van Camp et al., 2019			✓		✓		✓					✓	✓		✓				5						✓		✓					✓						✓	✓							✓			✓		✓
	Blease et al., 2019 *				✓		✓	✓	✓							✓				720						✓		✓				✓									✓						✓			✓		✓

The gray rows designate “design” studies. *Indicates that paper appears in more than one category.

Additionally, articles were classified by whether or not they described research surrounding a “real tool” that consisted of at least a working prototype involving the intended ML/AI, or research of “hypothetical tools,” that were either in the process of being designed or were described abstractly to participants for the purpose of conducting empirical research (see “Real Tool” columns in Table 3). This distinction was valuable when describing and comparing things like classes of algorithms and validation efforts.

The types of clinical AI tools described in the included articles can be defined as AI tools that assist with diagnosis (*n* = 18), treatment planning (*n* = 13), risk assessment (*n* = 9), ambient intelligence or telemonitoring (*n* = 7), natural language processing (*n* = 5), and administrative tasks (*n* = 5) (see Supplementary Table 1 for a description of articles).

Thirty articles were published by the product designers, including 24 that described a finished or nearly finished design that used actual ML algorithms, while six related product design teams detailed preliminary research conducted prior to design or completion of a prototype. Four studies were conducted by a third party after a product was implemented, and 10 were empirical research efforts that involved only hypothetical AI tools for the purpose of conducting research into clinicians’ experience with clinical AI tools, or their opinions, needs, or desires (see columns labeled “Type of study” and “Real tool” columns in Table 3).

A variety of algorithms were described, including hypothetical classes of products (e.g., “digital phenotyping” of psychiatric symptoms using biosensors, Bourla et al., 2020), specific types of algorithms (e.g., case based reasoning for diagnosis, Ehtesham et al., 2019), and existing proprietary tools (e.g., “Brilliant Doctor,” Wang et al., 2021) (see “Algorithms used” columns in Table 3).

**FIGURE 2:**
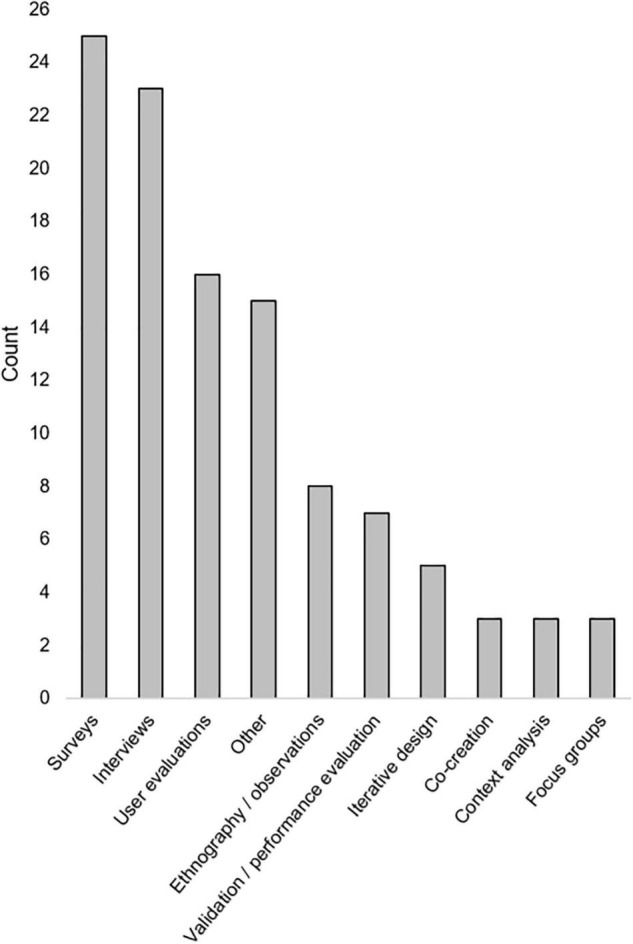
Methods used within included articles and counts.

**FIGURE 3:**
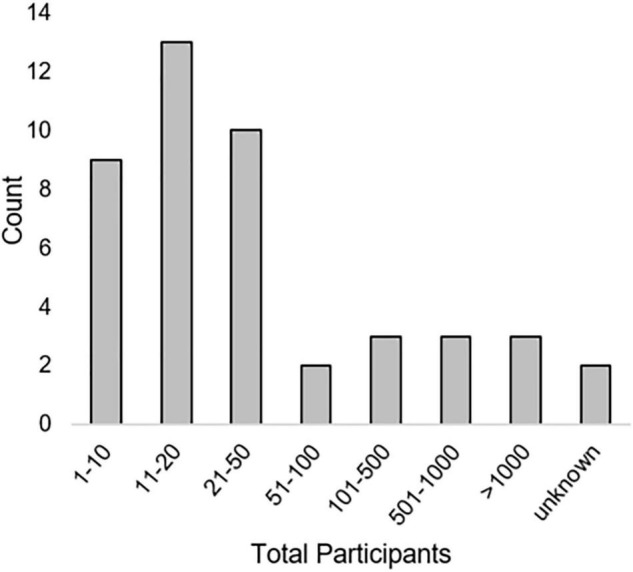
Total number of participants included in studies.

A variety of qualitative and quantitative research methods were described. The most used methods were interviews (*n* = 23) and various types of surveys (*n* = 23). Most articles (*n* = 39) included some measure of user opinions. Seventeen articles included some measure of performance or errors [see “Method type,” “Method(s),” and “Metric(s)” columns in Table 3]. Method types and the number of times they were employed in included articles is seen in Figure 2. Total numbers of participants included within studies described in included articles can be seen in Figure 3. While all articles included the consideration of clinicians as key stakeholders, nine also included patients as stakeholders, and eight included some other type of stakeholder [(e.g., hospital leaders, Sendak et al., 2020; or researchers, Nemeth et al., 2016); see “Stakeholders considered” columns in Table 3].

### Details on Design Studies

The 24 articles that were published by authors who were also the tools’ designers and were far enough into the design process to study a “real tool” (i.e., an actual clinical AI product was described and demonstrated to clinical end users) were our primary interest and are discussed further below (also see “Type of study” columns, and rows in gray in Table 3). As our goal in this review was to examine how clinical AI tool designers worked to integrate the needs of users, and how they measured the success of their designs, this subset of papers was critical for this examination.

#### Details on Algorithms

Of the 24 design articles, which included actual AI/ML, 10 used some type of deep learning, such as a Convolutional Neural Network (Tschandl et al., 2020) or deep Q-learning (Lee et al., 2020), or described a generic deep learning algorithm. Six provided a name for their tools or algorithms, possibly to reference them with regards to previous or future publications (e.g., “HealthXAI,” Khodabandehloo et al., 2021) and 11 specified other particular types of algorithms and/or sources of code (e.g., SVM with the scikit-learn python package, Lee et al., 2020). Six articles gave a generic description of the algorithm within the article, with three including references to other work further describing the algorithms (Brennan et al., 2019; Beede et al., 2020; Benrimoh et al., 2021) (see “Algorithms used” columns in Table 3).

#### How and When Are Users Consulted?

We determined what types of clinicians were the intended end users within included articles by examining explicit statements by authors as well as the individuals who were included as participants within studies. Of the 24 articles published by designers, most tools (*n* = 15) were intended for physician use. Four were intended for nurse use and six were intended for clinicians broadly. Twenty-three included qualitative measures, 20 included quantitative measures, with 19 including both. Five efforts consulted users throughout the design process (three or more times), and seven others consulted users at least two times during the process. Nine total articles reported designers’ efforts to engage users at the beginning, prior to any design (e.g., a needs assessment). Twelve only reported user feedback after the design process that was described in the article was completed (see “When were users consulted” columns in Table 3).

#### Stakeholders Included in Design

We considered “stakeholders” of a design to include individuals who were the intended users as well as other individuals who would be directly or indirectly affected by the clinical AI tools. We paid attention to any mention of any individuals besides end users (i.e., other stakeholders) whose needs and desires were considered during the design process. Clinical end users were considered as stakeholders in all included articles whether this was explicitly stated or not, and seven considered patients as stakeholders of the design. Only three included stakeholders other than clinicians or patients (e.g., administrators and care managers) in the design process.

#### Trust

Since the calibration of trust is an important feature that influences clinician adoption of clinical AI tools, we examined the ways in which these articles assessed clinician perceptions related to trust of clinical AI tools (e.g., assessments of the extent to which tool output was trusted, or discussion of what tool features and/or training, validation, and integration efforts could increase or calibrate trust in AI tools). Seven of the 24 articles included trust as a primary measure (i.e., trust was a measure within a survey or a question within an interview, etc.), and 12 total articles explicitly discussed clinician trust. Twelve articles did not discuss or evaluate whether users trusted tools.

#### Validation

Fifteen articles claimed to have validated their tool, either within the publication itself or in other articles published by the authors. Four articles reported measures of accuracy but did not report a comparison against that of clinical experts. Five articles did not report any type of validation within the paper or other currently published literature.

## Discussion

While clinical AI products are rapidly proliferating, our review shows that consultation with clinical end users prior to and throughout the design process is inconsistent. In addition, descriptions of the types of ML algorithms used in tools as well as model performance through validation and comparison to the performance of clinical experts (i.e., a “gold standard”) is not universal across efforts. The findings from this literature review shows that a human-centered design approach (i.e., commitment to end-user engagement throughout the design process) and attention to clinician trust through explicit evaluation and transparency of the ML used within the clinical AI tool are frequently underdescribed or not presented in published articles. This limitation makes it challenging for such efforts to demonstrate the comprehensive value of their tools to clinicians.

### How Design is Approached – “Worthy Nails”

The main purposes of this article is to better understand how designers of clinical AI tools determined what end users wanted and needed from these tools, how they incorporated feedback into their designs, and how they measured and reported. A key component of this success is ensuring that the design is focused on addressing specific clinical issues that are perceived as important to clinicians, which can only be determined through explicit conversation, and may be best realized through applying standardized research and design methodology. For instance, within our own research, we have found that the involvement of clinicians within large meetings can limit the opportunities for individuals to be heard or to elicit deep conversations about needs and challenges, particularly when supervisors are present or when the conversation focuses on meeting deadlines set by the institution. One method for achieving this goal is to involve the users in the beginning and throughout the design process through research activities (Kujala, 2010), especially as additional implicit and/or explicit needs can emerge over time. HCD methodology emphasizes the engagement of end users and other key stakeholders at the beginning and throughout the design process. Employing HCD methods provides designers with the opportunity to develop clinical AI tools that can meet the needs of clinical end users for successful implementation into healthcare (Beres et al., 2019), and can assist designers in gaining and appropriately calibrating clinician and patient trust in healthcare innovations (Glikson and Woolley, 2020; Wheelock et al., 2020; Benda et al., 2021), which is imperative for clinician adoption (Tcheng et al., 2017). Efforts to develop AI tools without first understanding the specific needs identified by intended users risk a “law-of-instrument” mentality, where, “I have a hammer, so let me treat all problems as nails” (Gellner and Kaplan, 1965). The mentality runs the risk of introducing cognitive bias into the process, whereby assumptions are made by the tool designers about the applicability of an AI solution to a particular problem, without deep understanding of the fundamental needs and challenges of users in that space. The pattern of fitting the problem to the tool rather than the tool to the problem is a known challenge in the development of clinical AI, partially due to the complexity of the development of ML algorithms and the sparse availability of rich datasets necessary for such development efforts (Wiens et al., 2019). We refer to these efforts as “worthy nails,” because while it is a worthy ambition to create tools to assist with important and persistent medical challenges, research into clinical AI tool success and failure suggests that the limited involvement of clinical end users throughout the process reduces the odds of successful implementation (Khairat et al., 2018; Yang et al., 2019). Our review findings indicate that end user involvement is inconsistent, or at least inconsistently reported on, and therefore highlights one potential solution for the challenges faced by healthcare organizations in successfully implementing clinical AI.

### What Does This Review Tell Us About the Current State of Artificial Intelligence in Healthcare?

The tools described by papers in this review can be categorized as tools that assist clinicians with diagnosis, treatment planning, risk prediction, ambient intelligence/telemonitoring, NLP, and administrative tasks. This review offers a “snapshot” of the current state of published literature on clinical AI tools that are designed for clinician use, which reveals that much more work is needed to establish consistent design and evaluation procedures for such tools to maximize their benefits within healthcare. While the number of articles published each year fitting our inclusion criteria increased over time, many efforts did not report any level of engaging of users in the tool design (e.g., the “NU” articles), although it is possible that some efforts were conducted but not reported. Additionally, while many of the design articles presented or claimed some form of model validation, there was no evidence of a universal commitment to comparing model performance to the “gold standard” of expert clinician performance. This finding is consistent with other recent literature (Sendak et al., 2019; Choudhury and Asan, 2020; Asan and Choudhury, 2021). However, it is also important to note here that validation efforts are necessary upon implementation of any clinical AI tool into a healthcare system, yet many of the innovations described in reviewed articles were not yet implemented into actual healthcare institutions. Others have discussed the disparity between studies reporting the design of new tools within a lab, and studies that present information about the real integration, evaluation, and redesign that occurs upon translation into real-world clinical environments (Sujan et al., 2019; Choudhury and Asan, 2020; Sendak et al., 2020; Osman Andersen et al., 2021).

### Limitations

This review is limited in a variety of ways. While this systematic approach attempted to capture all relevant work in the last 5 years, it is likely that some relevant articles were not discovered. The database search parameters were created to capture a broad range of publications, but also with a clear scope in mind, so as to limit the amount of ineligible articles requiring review. Another important limitation to note is that our categorization of the data from each effort described in the surveyed articles (e.g., at which point users were consulted, which stakeholders were included in design) only included information that was described within the articles themselves. It is possible, and even likely, that some design efforts included stakeholders and steps that were not described in the publications, therefore the authors of this review would not be aware of this data. Additionally, as the focus of this article was how users were integrated into the design process and the methodologies used, we did not focus on whether or not authors explicitly claimed to apply theoretical frameworks such as HCD. Nor were we able to report whether or not design teams included clinicians, or if clinicians co-authored articles, as this was not consistently reported on within articles. Additionally, since we were specifically interested in tools with clear research goals and outcomes, only peer-reviewed, primary research articles were included in the analysis, which may have excluded a number of related works.

### Research and Design Opportunities Discovered Through This Review

This review offers is a detailed comparison of the current clinical tasks and domains algorithmic tools are being applied to, and the research methods that are employed by tool designers to engage users and stakeholders within the design process. Our work compliments other recent studies that have identified a greater need for understanding clinical end users in the design process and the need for standardized validation and reporting of model performance (Middleton et al., 2016; Yang et al., 2019; Choudhury and Asan, 2020; Asan and Choudhury, 2021; Osman Andersen et al., 2021). This review also points to the need for a standardized protocol for design and implementation of clinical AI tools to ensure that they are helpful to clinicians upon implementation (Sujan et al., 2019; Wiens et al., 2019; Li et al., 2020; Osman Andersen et al., 2021). Additionally, we join with other authors (Cai et al., 2019b; Wheelock et al., 2020; Khodabandehloo et al., 2021), to suggest that a commitment to transparency in how tool output and specifications are presented to clinicians, and how this is reported on within the literature, should be a focus for designers and researchers.

Through our review and comparison, we have discovered that there may be opportunities to focus on nurses as clinical users, as the majority of studies included were developed for physicians. Additionally, the administrative burden associated with medical practice has been identified as a top contributor to clinician burnout (National Academies of Sciences, Engineering and Medicine et al., 2019). A relatively low number of articles included in our review are focused on assisting clinicians with administrative tasks (11%, *n* = 5/45 articles), and therefore designers may find that this is a domain with great research and design opportunities. It is our hope that other researchers may use our Product Matrix (Table 3) to quickly identify literature relevant to their own work to elucidate key considerations, and empower them in selection of appropriate human-centered methods that may be applied to their own work.

### Future Work: Integrating Human-Centered Design Into Development and Testing Phases of Artificial Intelligence in Healthcare

Based on our examination of the current literature, we argue that there are a number of investigative gaps that require more attention from clinical AI tool designers. In particular, it is critical to establish when and how to engage clinical end users and other key stakeholders in the design process, how to foster transparency of design and evaluated performance, and how to increase and appropriately calibrate clinician trust in clinical AI tools.

Future work will focus on establishing standard methodology that can be used by researchers to provide a strong, evidence based argument demonstrating the value of their tool to the intended clinical end users. The anticipated steps in this process include: (1) Determining the direct value of the tool for identified clinical end users and key stakeholders; (2) Establishing how the design and implementation of the clinical AI would be valuable to clinicians; (3) Verifying that tools are valuable and providing quantitative and qualitative evidence of the value; (4) Ensuring that tools add sustained (and adaptive) value for clinicians and key stakeholders once implemented into everyday clinical practice.

## Data Availability Statement

The original contributions presented in the study are included in the article/Supplementary Material, further inquiries can be directed to the corresponding author/s.

## Author Contributions

All authors listed have made a substantial, direct, and intellectual contribution to the work, and approved it for publication.

## Conflict of Interest

The authors declare that the research was conducted in the absence of any commercial or financial relationships that could be construed as a potential conflict of interest.

## Publisher’s Note

All claims expressed in this article are solely those of the authors and do not necessarily represent those of their affiliated organizations, or those of the publisher, the editors and the reviewers. Any product that may be evaluated in this article, or claim that may be made by its manufacturer, is not guaranteed or endorsed by the publisher.
