# Supplementary material for: Inclusion of Clinicians in the Development and Evaluation of Clinical Artificial Intelligence Tools: A Systematic Literature Review
Source: Front Psychol. 2022 Apr 7;13:830345. doi: 10.3389/fpsyg.2022.830345 (PMC9022040; doi:10.3389/fpsyg.2022.830345)
Supplement: Supplementary file 1 [file Table_1.docx]

Supplementary Material

Table 1: Articles included in review by product type, title, authors, year published, and journal

| Type of tool | Paper title | | Authors | Year published | Journal |  |
| --- | --- | --- | --- | --- | --- | --- |
| Diagnosis | A Decision Support System for Diagnostics and Treatment Planning in Traumatic Brain Injury. | | Umer et al | 2019 * | IEEE J Biomed Health Inform |  |
|  | A Human-Centered Evaluation of a Deep Learning System Deployed in Clinics for the Detection of Diabetic Retinopathy | | Beede et al | 2020 | CHI 2020 |  |
|  | Developing a new intelligent system for the diagnosis of oral medicine with case-based reasoning approach | | Ehtesham et al | 2019 | Oral Diseases |  |
|  | ECGLens: Interactive Visual Exploration of Large Scale ECG Data for Arrhythmia Detection | | Xu et al | 2018 | CHI 2018 |  |
|  | HealthXAI: Collaborative and explainable AI for supporting early diagnosis of cognitive decline | | Khodabandehloo et al | 2021 * | Future Gener Comput Syst |  |
|  | Human-Centered Tools for Coping with Imperfect Algorithms During Medical Decision-Making | | Cai et al | 2019 | CHI 2019 |  |
|  | A Cloud-based Platform for the Non-invasive Management of Coronary Artery Disease | | Sakellarios et al | 2020 * | Enterp Inf Syst |  |
|  | Human-computer collaboration for skin cancer recognition. | | Tschandl et al | 2020 | Nature medicine |  |
|  | NLPReViz: An Interactive Tool for Natural Language Processing on Clinical Text. | | Trivedi et al | 2018 * | JAMIA |  |
|  | "Brilliant AI Doctor" in Rural Clinics: Challenges in AI-Powered Clinical Decision Support System Deployment | | Wang et al | 2021 * | CHI 2021 |  |
|  | Implementation of artificial intelligence (AI) applications in radiology: hindering and facilitating factors | | Strohm et al | 2020 | European Radiology |  |
|  | Adapted Visual Analytics Process for Intelligent Decision-Making: Application in a Medical Context | | Ltifi et al | 2020 | Int J Inf Technol Decis Mak |  |
|  | Are student nurses ready for new technologies in mental health? Mixed-methods study. | | Bourla et al | 2020 * | Nurse Educ Today |  |
|  | Artificial Intelligence and the Future of Primary Care: Exploratory Qualitative Study of UK General Practitioners’ Views | | Blease et al | 2019 * | JMIR |  |
|  | "Hello AI”: Uncovering the Onboarding Needs of Medical Practitioners for Human–AI Collaborative Decision-Making | | Cai et al | 2019 | Proc. ACM Hum.-Comput. Interact |  |
|  | Impact of the rise of artificial intelligence in radiology: What do radiologists think? | | Waymel et al | 2019 | Diagn Interv Imaging |  |
|  | "It cannot do all of my work": Community Health Worker Perceptions of AI-Enabled Mobile Health Applications in Rural India | | Okolo et al | 2021 | CHI 2021 |  |
|  | The Integration of Artificial Intelligence in Medical Imaging Practice: Perspectives of African radiographers. | | Botwe et al | 2021 | Radiology |  |
| Treatment planning | A Decision Support System for Diagnostics and Treatment Planning in Traumatic Brain Injury. | | Umer et al | 2019 * | IEEE J Biomed Health Inform |  |
|  | CarePre: An Intelligent Clinical Decision Assistance System | | Jin et al | 2020 * | ACM Trans Comput Healthc |  |
|  | Co-Design and Evaluation of an Intelligent Decision Support System for Stroke Rehabilitation Assessment | | Lee et al | 2020 * | Proc ACM Hum Comput Interact |  |
|  | OrderRex clinical user testing: a randomized trial of recommender system decision support on simulated cases | | Kumar et al | 2020 | JAMIA |  |
|  | Revealing ICU Cognitive Work Through Naturalistic Decision-Making Methods. | | Nemeth et al | 2016 | J Cogn Eng Decis Mak |  |
|  | A Cloud-based Platform for the Non-invasive Management of Coronary Artery Disease | | Sakellarios et al | 2020 * | Enterp Inf Syst |  |
|  | TTTS-GPS: Patient-specific Preoperative Planning and Simulation Platform for Twin-to-Twin Transfusion Syndrome Fetal Surgery. | | Torrents-Barrena et al | 2019 | Comput Methods Programs Biomed |  |
|  | "Brilliant AI Doctor" in Rural Clinics: Challenges in AI-Powered Clinical Decision Support System Deployment | | Wang et al | 2021 * | CHI 2021 |  |
|  | Using a Simulation Centre to Evaluate Preliminary Acceptability and Impact of an Artificial Intelligence-Powered Clinical Decision Support System for Depression Treatment on the Physician-patient Interaction. | | Benrimoh et al | 2021 | BJPsych Open |  |
|  | Designing AI for Trust and Collaboration in Time-Constrained Medical Decisions: A Sociotechnical Lens | | Jacobs et al | 2021 | CHI 2021 |  |
|  | Investigating the Heart Pump Implant Decision Process: Opportunities for Decision Support Tools to Help | | Yang et al | 2016 | CHI 2016 |  |
|  | Unremarkable AI: Fitting Intelligent Decision Support into Critical, Clinical Decision-Making Processes | | Yang et al | 2019 | CHI 2019 |  |
|  | Artificial Intelligence and the Future of Primary Care: Exploratory Qualitative Study of UK General Practitioners’ Views | | Blease et al | 2019 * | JMIR |  |
| Type of tool | Paper title |  | | Year published | Journal | |
| Risk assessment |  |  | |  |  | |
|  | CarePre: An Intelligent Clinical Decision Assistance System | Jin et al | | 2020 * | ACM Trans Comput Healthc | |
|  | Comparing clinical judgment with the MySurgeryRisk algorithm for preoperative risk assessment: A pilot usability study. | Brennan et al | | 2019 | Surgery | |
|  | Integrating a Machine Learning System Into Clinical Workflows: Qualitative Study. | Sandhu et al | | 2020 | JMIR | |
|  | Real-World Integration of a Sepsis Deep Learning Technology Into Routine Clinical Care: Implementation Study | Sendak et al | | 2020 | JMIR medical informatics | |
|  | Realization of a Service for the Long-term Risk Assessment of Diabetes-related Complications. | Lagani et al | | 2015 | J Diabetes Complications | |
|  | Technology Acceptance of a Machine Learning Algorithm Predicting Delirium in a Clinical Setting: a Mixed-Methods Study | Jauk et al | | 2021 | J Med Syst | |
|  | Designing for Physician Trust: Toward a Machine Learning Decision Aid for Radiation Toxicity Risk | Gilbank et al | | 2020 | Ergon Des | |
|  | Are student nurses ready for new technologies in mental health? Mixed-methods study. | Bourla et al | | 2020 * | Nurse Educ Today | |
|  | Barriers to Implementing an Artificial Intelligence Model for Unplanned Readmissions. | Baxter et al | | 2020 | ACI open | |
| Ambient Intelligence and Telemonitoring | Acceptability Among Community Healthcare Nurses of Intelligent Wireless Sensor-system Technology for the Rapid Detection of Health Issues in Home-dwelling Older Adults. | Cohen et al | | 2017 | Open Nurs J | |
|  | Co-Design and Evaluation of an Intelligent Decision Support System for Stroke Rehabilitation Assessment | Lee et al | | 2020 * | Proc ACM Hum Comput Interact | |
|  | HealthXAI: Collaborative and explainable AI for supporting early diagnosis of cognitive decline | Khodabandehloo et al | | 2021 * | Future Gener Comput Syst | |
|  | Are student nurses ready for new technologies in mental health? Mixed-methods study. | Bourla et al | | 2020 * | Nurse Educ Today | |
|  | Conditions and ethical challenges that could influence the implementation of technologies in nursing homes: A qualitative study. | Bourbonnais et al | | 2019 | Int J Older People Nurs | |
|  | Improvements in patient monitoring in the intensive care unit: Survey study | Poncette et al | | 2020 | JMIR | |
|  | Psychiatrists' Attitudes Toward Disruptive New Technologies: Mixed-Methods Study | Bourla et al | | 2018 | JMIR mental health | |
| NLP | An Observational Study to Evaluate the Usability and Intent to Adopt an Artificial Intelligence–Powered Medication Reconciliation Tool | Long, 2016 | | 2016 * | Interact J Med Res | |
|  | Interactive NLP in Clinical Care: Identifying Incidental Findings in Radiology Reports. | Trivedi et al | | 2019 | Appl Clin Inform | |
|  | NLPReViz: An Interactive Tool for Natural Language Processing on Clinical Text. | Trivedi et al | | 2018 * | JAMIA | |
|  | Investigating the Barriers to Physician Adoption of an Artificial Intelligence- Based Decision Support System in Emergency Care: An Interpretative Qualitative Study. | Petitgand et al | | 2020 | Stud Health Technol Inform | |
|  | A Clinician Survey of Using Speech Recognition for Clinical Documentation in the Electronic Health Record. | Goss et al | | 2019 | Int J Med Inform | |
| Administrative Tasks | An Interactive Relevance Feedback Interface for Evidence-Based Health Care | Donoso-Guzmán et al | | 2018 | IUI | |
|  | An Observational Study to Evaluate the Usability and Intent to Adopt an Artificial Intelligence–Powered Medication Reconciliation Tool | Long et al | | 2016 * | Interact J Med Res | |
|  | Assisted Medication Management in Elderly Care Using Miniaturised Near-Infrared Spectroscopy | Klakegg et al | | 2018 | Proc ACM Interact Mob Wearable Ubiquitous Technol | |
|  | Development and Preliminary Evaluation of a Visual Annotation Tool to Rapidly Collect Expert-Annotated Weight Errors in Pediatric Growth Charts. | Van Camp et al | | 2019 | Studies in Health Technology and Informatics | |
|  | Artificial Intelligence and the Future of Primary Care: Exploratory Qualitative Study of UK General Practitioners’ Views | Blease et al | | 2019 * | JMIR | |
|  | * indicates that the paper appears in multiple categories |  | |  |  | |
